# Evaluation of Safety and Efficacy of Cell Therapy Based on Osteoblasts Derived from Umbilical Cord Mesenchymal Stem Cells for Osteonecrosis of the Femoral Head: Study Protocol for a Single-Center, Open-Label, Phase I Clinical Trial

**DOI:** 10.3390/ph17101366

**Published:** 2024-10-13

**Authors:** Seung-Hoon Baek, Bum-Jin Shim, Heejae Won, Sunray Lee, Yeon Kyung Lee, Hyun Sook Park, Shin-Yoon Kim

**Affiliations:** 1 Department of Orthopedic Surgery, Kyungpook National University Hospital, Daegu 41944, Republic of Korea; insidme@paran.com (S.-H.B.); hj8403@gmail.com (H.W.); 2 Department of Orthopedic Surgery, School of Medicine, Kyungpook National University, Daegu 41944, Republic of Korea; 3 Department of Orthopedic Surgery, College of Medicine, Yeungnam University, Daegu 42415, Republic of Korea; redpross@naver.com; 4 Cell Engineering For Origin (CEFO) Research Center, Seoul 03150, Republic of Korea; sunray@cefobio.com (S.L.); lyk412@cefobio.com (Y.K.L.)

**Keywords:** hip, osteonecrosis of the femoral head, core decompression, umbilical cord-derived mesenchymal stem cell, osteoblast, cell therapy, CF-M801

## Abstract

Although mesenchymal stem cells (MSCs) insertion has gained recent attention as a joint-preserving procedure, no study has conducted direct intralesional implantation of human umbilical cord-derived MSCs (hUCMSCs) in patients with ONFH. This is a protocol for a phase 1 clinical trial designed to assess the safety and exploratory efficacy of human umbilical cord-derived osteoblasts (hUC-Os), osteogenic differentiation-induced cells from hUCMSCs, in patients with early-stage ONFH. Nine patients with Association Research Circulation Osseous (ARCO) stage 1 or 2 will be assigned to a low-dose (1 × 10^7^ hUC-O cells, *n* = 3), medium-dose (2 × 10^7^ cells, *n* = 3), and high-dose group (4 × 10^7^ cells, *n* = 3) in the order of their arrival at the facility, and, depending on the occurrence of dose-limiting toxicity, up to 18 patients can be enrolled by applying the 3 + 3 escalation method. We will perform hUC-O (CF-M801) transplantation combined with core decompression and follow-up for 12 weeks according to the study protocol. Safety will be determined through adverse event assessment, laboratory tests including a panel reactive antibody test, vital sign assessment, physical examination, and electrocardiogram. Efficacy will be explored through the change in pain visual analog scale, Harris hip score, Western Ontario and McMaster Universities Osteoarthritis Index, ARCO stage, and also size and location of necrotic lesion according to Japanese Investigation Committee classification before and after the procedure. Joint preservation is important, particularly in younger, active patients with ONFH. Confirmation of the safety and efficacy of hUC-Os will lead to a further strategy to preserve joints for those suffering from ONFH and improve our current knowledge of cell therapy.

## 1. Introduction

Osteonecrosis of the femoral head (ONFH) usually affects young adults in their 30s to 50s and progresses to femoral head collapse in 60% of asymptomatic patients [1,2]. Total hip arthroplasty (THA) is an effective treatment for advanced ONFH with femoral head collapse but can cause complications such as infection and periprosthetic fracture and may require later revisions due to osteolysis or loosening [3,4,5]. Therefore, joint-preserving procedures must be considered, particularly in the treatment of early-stage ONFH before progression to head collapse, since ONFH often occurs in active, younger patients.

Core decompression is a joint-preserving procedure that can be performed for early-stage ONFH. It has been reported to reduce pressure within the femoral head and promote revascularization to induce bone formation and remodeling [6]. However, a previous study reported that 38% of patients with early-stage ONFH underwent THA at an average of 26 months after core decompression [7], and there has been a controversy regarding the effect of core decompression on early-stage ONFH in previous studies [8,9].

Mesenchymal stem cells (MSCs) insertion, which was used as an adjunctive technique to core decompression, has recently gained attention as a joint-preserving procedure for early-stage ONFH [10,11,12]. MSCs may cause an increase of local cell populations and promote vascular repair and angiogenesis, thereby effectively promoting osteogenesis in the femoral head [13,14]. Several studies have reported that combined core decompression with MSC transplantation in patients with early ONFH showed more satisfactory clinical outcomes and survival rates than core decompression alone [11,12,15]. However, bone marrow or adipocyte-derived MSCs have very diverse cell sourcing and processing strategies, and progenitor cells involved in angiogenesis or bone formation may be insufficient [16]. In comparison, human umbilical cord-derived MSCs (hUCMSCs) have been reported to have osteogenic potential similar to bone marrow- or adipose-derived MSCs and secrete larger amounts of transforming growth factor beta (TGF-β) and vascular endothelial growth factors (VEGFs) [17,18,19]. To date, no study has been conducted to evaluate the safety and efficacy of direct intralesional implantation of human umbilical cord-derived osteoblasts (hUC-Os), osteogenic differentiation-induced cells from hUCMSCs, as a cell therapy for ONFH. The present phase 1 clinical trial is to assess the safety and efficacy of hUC-O-based cell therapy in patients with early-stage ONFH.

## 2. Methods

### 2.1. Aims of This Study

The purpose of this study is to evaluate the safety and exploratory efficacy of hUC-Os. This is a phase I clinical trial in which safety is the main concern and will be determined through adverse event assessment, laboratory tests, vital sign assessment, a physical examination, and an electrocardiogram (ECG) test. In addition, we aim to assess the exploratory efficacy of hUC-Os by comparing subjective preoperative and postoperative patient-reported outcomes, specifically, the pain visual analog scale (VAS) [20], Harris hip score (HHS) [21], and Western Ontario and McMaster Universities Osteoarthritis Index (WOMAC) [22]. Also, we aim to assess the radiographic results after hUC-O-based cell therapy using simple radiography and magnetic resonance imaging (MRI). The preoperative and postoperative the Association Research Circulation Osseous (ARCO) stage [23], necrotic lesion size [24,25], and Japanese Investigation Committee (JIC) stage [26] will be compared.

### 2.2. Study Design

The present study is an investigator-sponsored, prospective, single-institution, open-label, phase 1 clinical trial and has been approved by the institutional review board (IRB) of Kyungpook National University Hospital (IRB No. KNUH 2021-07-015) and Korea Food and Drug Administration (No. 10052) on 1 July 2021. The trial was registered at the Korean Clinical Trials Register (KCT0006627) on 30 September 2021.

### 2.3. Study Population

Participants must fulfill all of the following inclusion criteria to qualify for study enrollment: (1) male and female between the age of 19 and 70; (2) radiographically diagnosed with ONFH; (3) ARCO stage 1 or 2 of the classification criteria for ONFH; (4) symptoms persistent after conservative treatment including medication with duration of at least 3 months; and (5) have voluntarily consented to participate in this clinical trial. Exclusion criteria are as follows: (1) a person with femoral head collapse due to rapidly progressing ONFH; (2) clinically significant abnormal values in a screening test (aspartate aminotransferase (AST) or alanine transaminase (ALT) levels are 2.5 times or more of the upper normal limit specified by the clinical laboratory); (3) malignant bone tumor or bone metastasis in the proximal femur; (4) previous history of malignant tumors (but can be included if there was no relapse within 5 years after completing a treatment); (5) diagnosed with inflammatory arthritis around the femoral head; (6) diagnosed with acetabular dysplasia with a lateral center-edge angle <20°; (7) post-traumatic ONFH; (8) systemic or local infectious disease requiring systemic antibiotic treatment; (9) diagnosed with human immunodeficiency virus (HIV), hepatitis infection; (10) requires continuous, systemic, and high-dose corticosteroid therapy (prednisone administration at 7.5 mg/day or more) or administration of immunosuppressant within 6 months after core decompression; (11) stem-cell therapy for ONFH before screening; (12) heavy alcoholism and overconsumption within 6 months of screening; (13) have undergone core decompression or other treatments or procedures that can affect the femoral head; (14) body mass index ≥ 40 kg/m^2^ at the screening visit; (15) females with a possibility of pregnancy and do not agree to use medically approved contraceptive methods during the study period; (16) pregnant or breastfeeding; (17) received an investigational new drug from another clinical trial within 4 months before participating in this study; and (18) when the investigator determines that the subject is not suitable to participate in this clinical trial.

Of the patients who consent to participate in this clinical trial, those who satisfy the inclusion and exclusion criteria will be assigned to a low-dose (*n* = 3), middle-dose (*n* = 3), and high-dose group (*n* = 3) in the order of their arrival at the facility without random allocation and followed up for 12 weeks. Nine patients will be assigned if no dose-limiting toxicity (DLT) develops up to the highest dose. Depending on the occurrence of DLT, up to 18 patients can be enrolled by applying the 3 + 3 escalation method.

### 2.4. Characteristics and Preparation of the hUC-Os (CF-M801)

The bone-targeted cell therapy, named CF-M801 by developer (CEFO Co., Seoul, South Korea), was produced by inducing osteogenic differentiation of hUCMSCs into hUC-Os using an approved manufacturing method in a factory approved by regulatory agencies. CF-M801 has multiple functions in bone regeneration and angiogenesis and is characterized by the expression of cluster of differentiation (CD) markers [27,28] (Table 1).

CF-M801 consists of 10 million cells/mL and is supplied in frozen form in accordance with the Investigational Product (IP) management policy. An off-white cell suspension is stored under cryogenic conditions (≤−135 °C) and can be used for 1.6 years from the date of manufacture. Vials are transported in a portable freezer prior to use, thawed for 3 to 5 min at 35–37 °C, and must be used within 2 h after thawing. A syringe is filled with the whole volume of cell suspension, shaken to achieve an even cell suspension before administration. A vial has a volume of 1 mL and contains 1 × 10^7^ hUC-Os. In this trial, CF-M801 will be administered into the necrotic lesion of ONFH at a low (1 vial, 1 × 10^7^ cells), middle (2 vials, 2 × 10^7^ cells), or high dose (4 vials, 4 × 10^7^ cells) in the order of group allocation.

### 2.5. Procedure

Core decompression restores normal blood circulation within the femoral head by drilling a tunnel at the site of necrosis [6]. It reduces the increased intramedullary pressure and pain by promoting revascularization within the necrotic site [9]. In this clinical trial, a competent researcher or a designated physician will perform core decompression and administer CF-M801 through the following steps.

**Figure 1:**
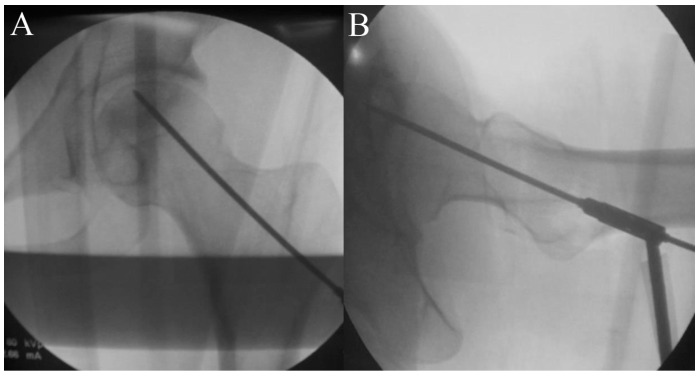
A guide pin is inserted under fluoroscopic guidance towards the necrotic area for a core tract. (**A**) Anterior posterior view. (**B**) Lateral view.

**Figure 2:**
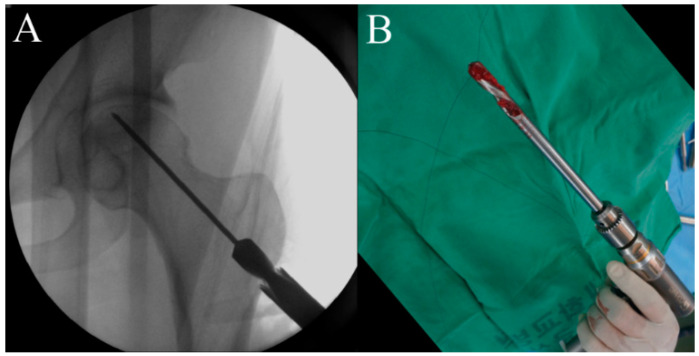
To guide a following hollow reamer, an entry hole is created using a cannulated solid reamer along the guide pin (**A**), and an autogenous bone chip is collected during reaming (**B**).

**Figure 3:**
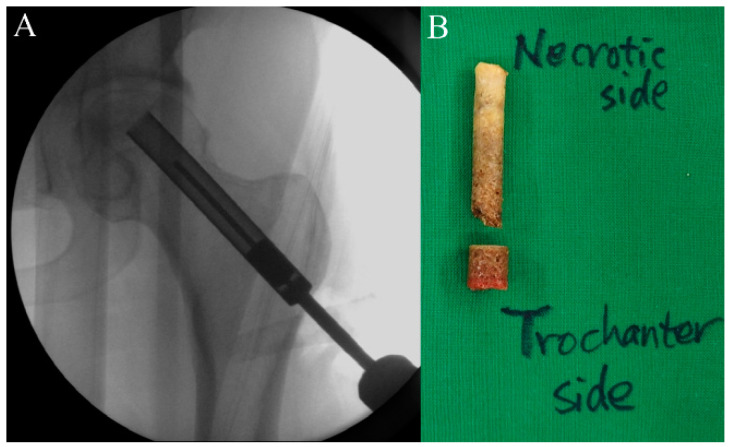
A core tract is created for core decompression using a hollow biopsy cannula (**A**). During this procedure, a cylindrical autogenous bone block is collected, of which a proximal necrotic portion will be sent for pathologic evaluation and a distal viable portion will be implanted as a bone plug after stem cell insertion (**B**).

**Figure 4:**
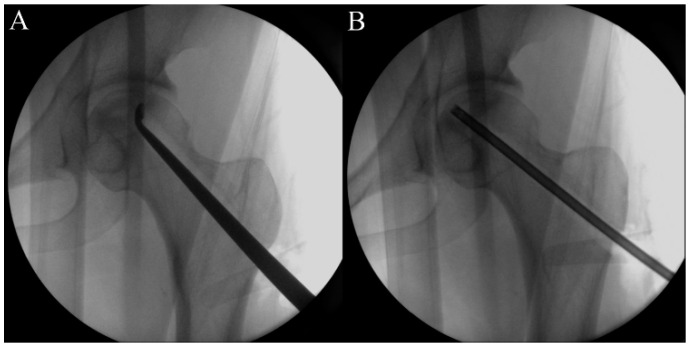
A curette is inserted to remove the necrotic lesion (**A**), followed by washing the necrotic bone debris (**B**).

**Figure 5:**
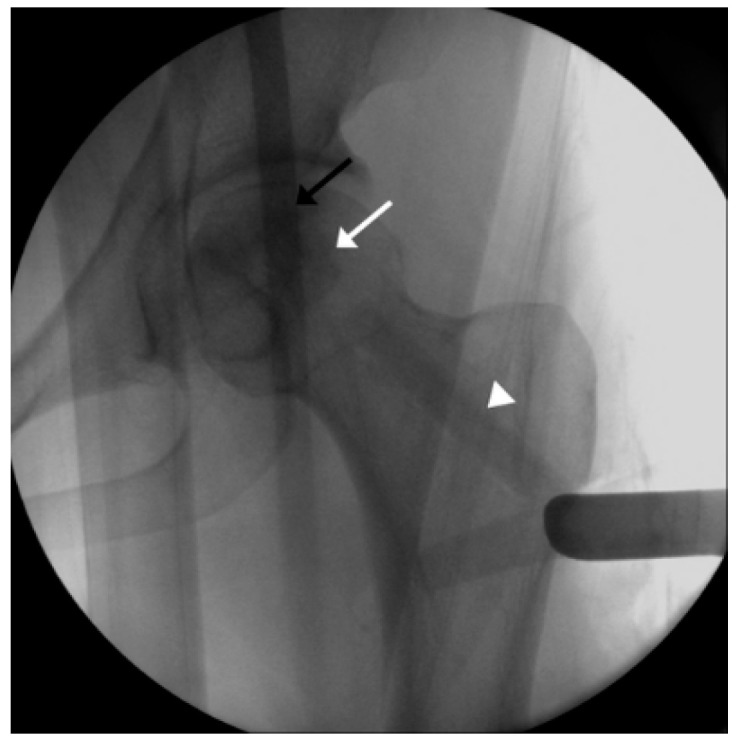
A mixture of cell and collagen putty is implanted into the lesion (black arrow), and the autogenous bone chip and cylindrical bone block is inserted into the remaining space in the core tract (white arrow). If there is significant space remaining in the distal portion of the core tract, a cylindrical hydroxyapatite and tri-calcium phosphate block may be inserted (arrowhead).

First, the size and location of the lesion will be checked before the procedure, and the patient will be placed on the fracture table. Then, a lateral small incision will be made, and a guide pin will be inserted for a core tract under fluoroscopic guidance (Figure 1). Next, an entry hole will be created using a cannulated solid reamer along the pin to guide a following hollow reamer, and an autogenous bone chip (which will be inserted distal to a mixture of hUC-Os) will be collected (Figure 2). Then, a core tract will be created using a hollow biopsy cannula with a diameter of 8–10 mm along the entry hole reaching to necrotic lesion, and a cylindrical autogenous bone block (which will be inserted as a bone plug following inserting autogenous bone chip) will be collected (Figure 3). Curettage will be performed to remove the necrotic bone, and the necrotic bone debris will be washed (Figure 4). During preparation of the recipient site, CF-M801 will be attached with collagen putty using fibrin glue. Collagen putty contains hydroxyapatite (HA) and tri-calcium phosphate (TCP) and can be identified under the fluoroscopic guidance. After the mixture of cell and collagen putty is brought to the necrotic lesion carefully under the fluoroscopic guidance, the remaining space in the core tract will be filled distally with the autogenous bone chip and cylindrical bone block collected in the previous steps. If there is significant space remaining in the distal portion of the core tract, a cylindrical HA and TCP block may be inserted (Figure 5). Gradual partial weight-bearing will be started at 6 weeks, and full weight-bearing walking will be permitted at 3 months postoperatively.

### 2.6. Timeline and Study Protocol

After a volunteer provides written consent to participate in the clinical trial, necessary examinations and tests will be performed within 3 weeks before CF-M801 administration (Visit 1) according to the study protocol (Table 2). Participants who meet the inclusion/exclusion criteria in the eligibility assessment will be subjected to core decompression and CF-M801 administration (Visit 2). Participants will be checked for acute adverse events for 3 days after administration of CF-M801 and will visit the facility after 2 (Visit 3), 6 (Visit 4), and 12 (Visit 5) weeks for a safety assessment. The safety assessment will be performed based on the same schedule for all participants, with only the administration dose varying.

To determine the maximal tolerated dose (MTD) of CF-M801, three participants will be sequentially allocated to a group starting with the low-dose group (1 × 10^7^ cells) according to the standard 3 + 3 dose escalation scheme [29]. The first participants in each dose group will be monitored for acute adverse events including DLT for 3 days after CF-M801 administration. DLT refers to when adverse events related to CF-M801 with grade ≥3 are observed based on the definition from the National Cancer Institute—Common Toxicity Criteria (NCI-CTC) version 5.0 [30]. Another two participants will be assigned to the group when DLT does not occur. Up to 18 patients can be enrolled, depending on the development of DLT. The decision process is as follows: (1) If none of the three participants experiences DLT, the dose will be increased to the next level. (2) If one of the three participants experiences DLT, three participants will be additionally assigned to the same dose group. (3) If one of the six participants (initial three + additional three participants) experience DLT, the dose will be increased to the next level. (4) However, if at least two of the six participants experience DLT, dose escalation will be canceled, and the MTD will become the previous dose level. (5) If at least two of the three participants experience DLT, dose escalation will be canceled, and the MTD will become the previous dose level. (6) If one of the three additional participants show DLT, no additional participants will be assigned to the dose group, and current participants will be monitored for safety.

Participants who will not complete the DLT assessment due to reasons independent of DLT will be deemed an inadequate basis for making decisions regarding dose escalation and an MTD assessment and can be replaced by participants who will be added to the same dose group.

### 2.7. Safety Assessment

Clinically significant abnormal findings from screening or before CF-M801 administration (during a screening test) will be recorded as medical history, and those that satisfy the definition of adverse events following CF-M801 administration will be recorded as adverse events. The safety assessment will be performed in accordance with the study protocol.

#### 2.7.1. Adverse Event

Participants will be instructed to report any adverse events, and researchers will examine participants for adverse events through regular or additional visits, interviews, and their history. DLT will be included as an adverse event. Any adverse events observed will be reported to the IRB throughout the study period.

#### 2.7.2. Laboratory Tests

Laboratory tests will be performed for all participants according to the clinical trial schedule to assess the overall health condition of participants. Participants will be instructed to fast (not allowed to drink beverages except for water or eat food within 8 h before the test) before visiting the facility for blood collection in preparation for laboratory tests. For screening tests (Visit 1), test results obtained in the last 4 weeks may be used instead. One retest may be performed at the discretion of the researcher during the screening period. If a retest has been performed, the results of the retest will be used to determine whether the participant meets the final inclusion and exclusion criteria. The following parameters will be assessed during the laboratory tests.

(1) Blood test: red blood cell (RBC) count, hemoglobin, hematocrit, platelet count, white blood cell (WBC) count, WBC differential count (neutrophil, lymphocyte, monocyte, eosinophil, and basophil), and erythrocyte sedimentation rate.
(2) Blood chemistry test: total protein, albumin, total bilirubin, AST, ALT, gamma-glutamyl transpeptidase, blood urea nitrogen, creatinine, glucose, alkaline phosphatase, total cholesterol, triglyceride, uric acid, C-reactive protein, panel reactive antibody (PRA), virus infection test (HIV antigen/antibody, hepatitis B surface antibody, anti-hepatitis C virus antibody, venereal disease research laboratory (VDRL).
(3) Urinalysis: protein (albumin), glucose, ketones, WBC, RBC.

Among the blood chemistry tests for viral infections, HIV antigen/antibody, hepatitis B antibody, anti-hepatitis C antibody, and VDRL tests will be performed at Visit 1. Allogeneic immune reaction will be validated through panel reactive antibody (PRA) test at Visit 1 and Visit 5 (Week 12).

#### 2.7.3. Vital Signs

For vital signs, blood pressure, pulse, respiratory rate, and body temperature will be measured. Vital signs will be measured before the scheduled test for each visit in accordance with the clinical trial schedule. Participants will be directed to relax for 5 min and be seated during the measurement.

#### 2.7.4. Physical Examination

Each participant will be physically examined through inspection, palpation, percussion, and auscultation in accordance with the clinical trial schedule to check their health conditions and find any adverse events. Physical examination includes examination of the external appearance, head and neck, chest and lungs, heart, abdomen, urinary and reproductive systems, limbs, musculoskeletal system, nervous system, and lymph nodes.

#### 2.7.5. ECG Test

An ECG test will be performed for all participants at Visits 1, 3, 4, and 5. The examiner will determine whether the ECG results are normal or abnormal. If the results are abnormal, the examiner will record the clinical significance of the findings on the case report form (CRF).

### 2.8. Clinical Assessment

#### 2.8.1. Pain VAS Assessment [20]

Participants will mark their subjective pain level around the hip joint on a scale that ranges from “no pain (0)” to “severe pain (10)”. The examiner will determine the number corresponding to the point marked on the scale and record it to one out of 10 decimal places. To examine the changes in pain VAS at different time points after CF-M801 administration, pain VAS assessment will be performed at Visits 2 and 5.

#### 2.8.2. HHS Assessment [21]

HHSs will be assessed at Visits 2 and 5 to examine the participants’ hip joint function. The highest possible score is 100, and the following domains will be assessed: pain (44 points), gait function (11 points), support devices (11 points), distance walked (11 points), stairs (4 points), putting on shoes (4 points), sitting (5 points), entering public transportation (1 point), presence of deformity (4 points), and hip joint range of motion (5 points).

#### 2.8.3. WOMAC Score Assessment [22]

WOMAC scores will be assessed at Visits 2 and 5 to examine the changes in WOMAC scores after CF-M801 administration and assess participants’ joint function. WOMAC assesses three domains, namely, pain, stiffness, and physical function.

### 2.9. Radiologic Assessment

#### 2.9.1. Measuring Changes in Necrotic Lesion Size

To examine changes in the size of a necrotic lesion in the femoral head following CF-M801 administration, the modified Kerboul index will be measured on mid-coronal and mid-sagittal MRI images acquired during Visits 1 and 5 [24,25].

#### 2.9.2. ARCO Classification [23]

The ARCO classification is an improved version of the Ficat and Arlet classification based on the presence and severity of femoral head collapse and Steinberg classification based on lesion size and progression, which are commonly used to stage ONFH. Two independent examiners will analyze radiographs and MRI of the hip joint during Visit 1 and 5 to examine the changes in the ARCO stage following CF-M801 administration. They will then classify the patients who show changes in the ARCO stage following CF-M801 administration and femoral head collapse using the ARCO classification. Femoral head collapse corresponds to ARCO stage 3.

#### 2.9.3. JIC Classification [26]

The JIC classification classifies ONFH based on the location of the lesion. It is a radiographic classification method with three major types of ONFH and is based on the location of the lesion in respect to the weight-bearing surface. Two independent examiners will examine the changes in the JIC stage after CF-M801 administration by evaluating radiographs and MRI of the femoral region during Visit 1 and 5 and classify the lesions according to the JIC classification.

### 2.10. Data Collection, Management, and Analysis

#### 2.10.1. Data Collection and Management

Informed consent will be obtained from all participants, and all data will be collected according to the visit schedule in Table 2. Any missing values of safety and efficacy data will not be replaced, and, if the subject’s visit date falls outside the planned tolerance range (visit window), the measurement values for that visit will be treated as missing values. The CRFs will be the primary data collection tool for this trial, and all data requested on the CRFs must be recorded. An electronic data capture system (iMediData) will be used to submit data. Information related to trial participants will be kept confidential and stored securely in a separate space with a locking device, only accessible to research personnel. Trial records will be identified only by a coded participant number, and all records that contain the participants’ names or any identifying information will be stored separately. All local databases used for storage of trial data will be password-protected. 

#### 2.10.2. Data Analysis

This clinical trial was planned as a phase 1 clinical trial using a 3 + 3 dose escalation method, and, because three subjects will be assigned to each group, it is not appropriate for statistical processing. Thus, data analysis will be conducted in the following manner. For the data collected in this trial, continuous data will be presented as descriptive statistics (number of patients, mean, standard deviation, minimum, median, maximum) and categorical data will be presented as frequencies and percentages.

## 3. Discussion

Cell therapy is becoming more popular as a means to restore deficient local cell populations due to disease or injury. ONFH is associated with a decrease in progenitor cells in the femoral head that can influence bone repair due to alteration of intramedullary vascularity [31,32]. MSCs are multipotent cells that can differentiate into fibroblastic, osteogenic, myogenic, adipogenic, and reticular cells [32,33] and are mainly harvested from bone marrow, adipose tissue, and umbilical cord. Most of previous studies used bone marrow-derived MSCs (BMSCs) and showed satisfactory results [11,12,15,34,35]. A previous study with the longest follow-up duration of 25 years, which administered BMSCs to one side while performing core decompression alone to opposite side in 125 patients with bilateral ONFH, reported that the BMSC group had significantly lower THA conversion rates than the core decompression alone group (24% vs. 76%, *p* < 0.001) [15]. However, considering the natural course of ONFH according to the location and size of the lesion [1], the study was limited because it did not analyze the size and location of lesion before the procedure and it is difficult to evaluate the accurate effect of the BMSCs. Moreover, according to a recent systemic review, standardization is still insufficient for the collection, cell-processing, and delivery method of MSCs [16].

In animal experiments, hUCMSCs had an effect applicable to ONFH treatment. Kuang et al. [36] reported that hUCMSCs inhibited osteocyte apoptosis in steroid-induced ONFH rat models, and You et al. [19] showed that hUCMSCs promoted angiogenesis in a rat bronchopulmonary dysplasia model. In canine experiments comparing hUCMSCs with MSCs derived from adipose tissue and bone marrow, hUCMSCs showed higher osteogenic potential than other cell sources [18]. To the best of our knowledge, there has been one study that applied hUCMSCs to ONFH patients. Chen et al. [37] performed ‘intra-arterial’ injection of hUCMSCs on nine patients with ONFH at ARCO stage 2 to 3A and reported increased oxygen delivery at 3 days and reduced extent of ONFH at 2 years postoperatively. However, intra-arterial injection requires a higher dose than direct implantation into the lesion, and safety has not been established yet [13].

This study is the first clinical trial conducted for treatment of early-stage ONFH by direct intralesional application of hUC-O. In addition, this study will assess the clinical outcomes and evaluate the extent of necrotic lesions using MRI before and after treatment. We are also fully aware of the limitations of this study. First, this study is non-blinded and may have selection and detection biases. Second, this study is limited because of the short follow-up duration of 12 weeks. Indeed, 12 weeks are too short to conclude the efficacy of new treatment. However, this is a phase I clinical trial in which safety is the main concern and which tests the adverse effects, best dose, and timing of a new treatment [38]. The main objectives of this study are to evaluate any adverse events and to confirm the safety of administered hUC-Os through vital signs, physical examination, ECG tests, and laboratory tests, including PRA tests, to monitor allograft immune reactions following allogeneic cell transplantation [39]. Since the exploratory efficacy is included in this study as well, the minimum observation period of 12 weeks was required from the Korea Food and Drug Administration. In addition, long-term follow-up to 5 years will be conducted on the subjects enrolled, and all data will be submitted to the Korea Food and Drug Administration as well.

Nevertheless, we believe that this phase I clinical trial to confirm the safety and evaluate exploratory effects of hUC-Os on early-stage ONFH will lead to improve our current knowledge of cell therapy and add a new strategy to preserve joints for those suffering from ONFH.

## Figures and Tables

**Table 1:** Gross characterization of the cell therapy.

	Hematopoietic		Mesenchymal		Osteoblast		Angiogenesis
Markers	CD31	CD45	CD73	CD105	RUNX2	COL1A	ANGPT-1
Expression	≤5%	≤5%	≥50%	≥50%	≥5 copies	≥3000 pg/mL	≥2000 pg/mL

**Table 2:** Protocol and timeline for outcome assessments.

Period	Screening	Treatment ^(g)^	Follow-Up 1	Follow-Up 2	Study Completion /Dropout ^(i)^
Visit	1	2	3	4	5 ^(i)^
Week	Within-3	0	2	6	12
Visit window (days)	-	Within-3	±3	±7	+7
Written consent form ^(a)^	X				
Demographic information	X				
Body weight and height measurement	X				
Review medical/operation history	X	X ^(h)^			
Review medication history	X	X ^(h)^			
Vital signs ^(b)^	X	X ^(h)^	X	X	X
Physical examination	X	X ^(h)^	X	X	X
Clinical laboratory tests ^(c)^	X ^(C−1), C−2)^		X	X	X ^(C−1)^
ECG test	X		X	X	X
Pregnancy test ^(d)^	X				X
Radiographic examination	X ^(e)^		X	X	X
MRI examination	X				X
ARCO classification	X				X
JIC classification	X				X
Review inclusion/exclusion criteria	X	X			
Patient selection		X			
Group allocation		X			
Core decompression		X			
hUC-Os administration		X			
Pain VAS assessment		X ^(h)^			X
HHS assessment		X ^(h)^			X
WOMAC assessment		X ^(h)^			X
Adverse events assessment		X ^(f)^	X	X	X
Check concomitant drug use		X	X	X	X

ECG, electrocardiogram; MRI, magnetic resonance imaging; ARCO, Association Research Circulation Osseous; JIC, Japanese Investigation Committee; hUC-Os, human umbilical cord-derived osteoblasts; VAS, visual analog scale; HHS, Harris hip score; WOMAC, Western Ontario and McMaster Universities. ^(a)^ Written consent can be obtained from participants before the screening visit. ^(b)^ For vital signs, pulse, blood pressure, and body temperature will be measured. At Visit 2, vital signs will be measured before hUC-O administration. ^(c)^ For screening tests, test results obtained within 4 weeks after a visit can be used. A retest may also be performed once at the discretion of a researcher during the screening period. The following parameters are assessed during the laboratory tests. Blood test: red blood cell (RBC) count, hemoglobin, hematocrit, platelet count, white blood cell (WBC) count, WBC differential count (neutrophil, lymphocyte, monocyte, eosinophil, and basophil), and erythrocyte sedimentation rate; Blood chemistry test: total protein, albumin, total bilirubin, aspartate aminotransferase or alanine transaminase, gamma-glutamyl transpeptidase, blood urea nitrogen, creatinine, glucose, alkaline phosphatase, total cholesterol, triglyceride, uric acid, C-reactive protein, panel reactive antibody ^(C−1)^, virus infection test (human immunodeficiency virus antigen/antibody, hepatitis B surface antibody, anti-hepatitis C virus antibody, venereal disease research laboratory) ^(C−2)^; Urinalysis: protein (albumin), glucose, ketones, WBC, RBC. ^(d)^ A serum or urine human chorionic gonadotropin test will be performed for women of childbearing age except for those who have been sterilized or are menopausal. Women of childbearing age refer to women who have had their first period, have not been successfully surgically sterilized (via hysterectomy, bilateral tubal ligation, or bilateral oophorectomy), and are not menopausal. Menopause is defined as not having a period for >12 months after the final menstrual period. ^(e)^ Test results obtained within 4 weeks after screening can be used. ^(f)^ Acute adverse events that occur within 3 days after hUC-O administration will be investigated. ^(g)^ Patients will be hospitalized starting 1 day before hUC-O administration and will be monitored throughout the hospitalization period for adverse events. ^(h)^ Performed within 3 days and restrict intake of analgesics within 1 day before hUC-O administration. ^(i)^ For participants who were withdrawn following the group allocation, the procedures scheduled for the last visit will be performed.

## Data Availability

Data are contained within the article.

## Abbreviations

ALP	Alkaline phosphatase
ALT	Alanine aminotransferase
ARCO	Association research circulation osseous
AST	Aspartate aminotransferase
BMSC	Bone marrow-derived mesenchymal stem cell
CD	Cluster of differentiation
DLT	Dose-limiting toxicity
ECG	Electrocardiogram
HA	Hydroxyapatite
HHS	Harris hip score
HIV	Human immunodeficiency virus
hUC-O	Human umbilical cord-derived osteoblast
hUCMSC	Human umbilical cord-derived mesenchymal stem cell
IRB	Institutional review board
JIC	Japanese investigation committee
MRI	Magnetic resonance imaging
MSC	Mesenchymal stem cells
MTD	Maximal tolerated dose
NCI-CTC	National Cancer Institute—Common Toxicity Criteria
ONFH	Osteonecrosis of the femoral head
PRA	Panel reactive antibody
RBC	Red blood cell
TCP	Tri-calcium phosphate
TGF	Transforming growth factor
THA	Total hip arthroplasty
VAS	Visual analog scale
VEGF	Vascular endothelial growth factor
WBC	White blood cell
WOMAC	Western Ontario & McMaster Universities
